# Analysis of choroidal thickness on optical coherence tomography in a patient with sudden‐onset bilateral myopia, macular striae, and shallow anterior chamber after topiramate use

**DOI:** 10.1002/ccr3.7388

**Published:** 2023-05-20

**Authors:** Kishore Kumar Vatwani, Koushik Tripathy, Rohit Agarwal, Gopal Bandyopadhyay

**Affiliations:** ^1^ Department of Ophthalmology ASG Eye Hospital Kolkata West Bengal India; ^2^ Department of Vitreoretina and Uvea ASG Eye Hospital Kolkata West Bengal India

**Keywords:** anterior rotation of the ciliary body, choroidal effusion, ciliary effusion, secondary angle‐closure glaucoma, sulfa drugs

## Abstract

This patient presented with sudden onset myopia, shallow anterior chamber, and radial macular folds in both eyes after using topiramate. Ocular parameters including increased choroidal thickness normalized after cessation of topiramate.

A 17‐year‐old male presented with a sudden‐onset decline in uncorrected visual acuity (6/60 in the right eye/RE and 5/60 in the left eye/LE). There was no history of ocular redness or pain. Best‐corrected visual acuity was 6/6 in both eyes (−3.5/−0.5 × 90° RE and − 4 LE). The anterior chamber (AC) was shallow and intraocular pressure was 16 mm of Hg in both eyes. He had been taking topiramate tablet 25 mg twice daily (for epilepsy) for 2 weeks. An ophthalmic prescription 1 month back had documented normal anterior chamber depth, normal optic disc and macula, and uncorrected visual acuity of 6/6. The crystalline lens was clear in both eyes. Radial macular internal limiting membrane striae (Figure 1) were noted. A diagnosis of acute onset myopia and macular striae secondary to topiramate was made. Myopia and retinal folds resolved after 2 days of cessation of topiramate and starting of levetiracetam 500 mg twice daily (Figure 2). The anterior chamber depth also normalized. The subfoveal choroidal thickness on optical coherence tomography (OCT) reduced from 404 microns (Figure 3A) at presentation to 307 microns (Figure 3B) in the RE and 341 microns (Figure 3C) at presentation to 273 microns (Figure 3D) in LE.

**FIGURE 1:**
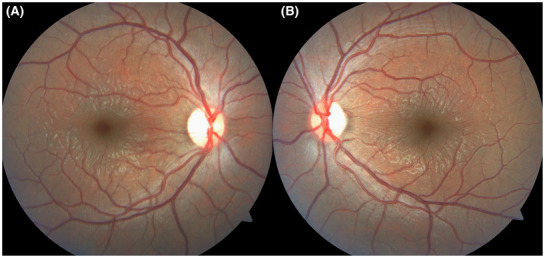
Fundus photo at the presentation of the right (A) and left (B) eye showing radial macular striae around the fovea.

**FIGURE 2:**
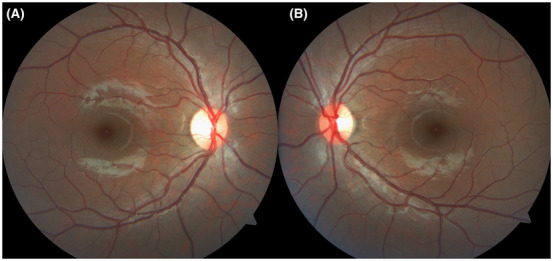
After stopping topiramate, the macular striae vanished in the right (A) and the left (B) eye.

**FIGURE 3:**
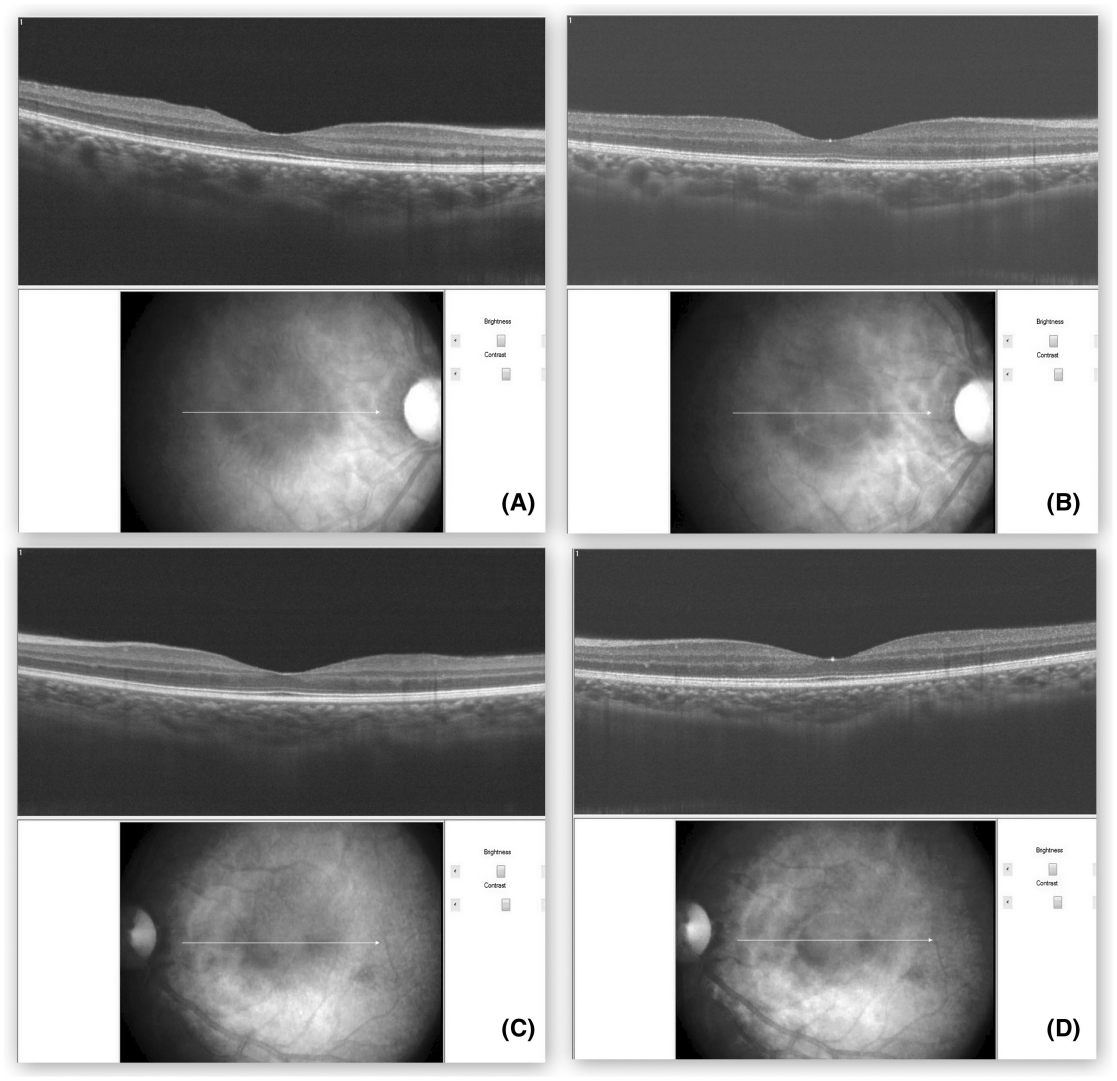
The subfoveal choroidal thickness on optical coherence tomography reduced from 404 microns (A) at presentation to 307 microns (B) after stopping topiramate in the right eye and 341 microns (C) at presentation to 273 microns (D) after stopping topiramate in the left eye.

Shallow AC in acute onset myopia hints toward the anterior movement of the lens and is a known adverse event after topiramate use. Topiramate causes ciliary and choroidal effusion and anterior rotation of the ciliary body.^1^ Some cases develop secondary angle closure glaucoma responding to topical atropine rather than peripheral iridotomy.^1^ Posterior segment adverse events include retinal striae, macular pigmentary changes, subretinal fluid,^1^ choroidal folds, choroidal detachment, and vitritis.^2^ The etiology of macular striae after topiramate use may involve choroidal effusion or traction over the macula due to forward traction on the vitreous body caused by the forward shift of the iris‐lens diaphragm. The retinal striae usually resolve after stopping topiramate though systemic steroid has also been tried.^3^ The drugs causing ciliary and choroidal effusion and secondary anterior rotation of the ciliary body include sulfa‐based drugs (topiramate, acetazolamide, methazolamide, indapamide, furosemide, cotrimoxazole, dapsone, sulfadiazine, hydrochlorothiazide, sulfonylureas, triptans, probenecid), spironolactone, promethazine, metronidazole, quinine, penicillamine, aspirin, and isotretinoin. The limitation of this report includes the absence of ultrasound biomicroscopy to detect anterior rotation of the ciliary body and ultrasonography to evaluate for choroidal effusion. However, the OCT showed increased choroidal thickness at the presentation with dilation of medium and large choroidal vessels. It is known that drug‐induced ciliary effusion can cause myopia without causing glaucoma or a rise in intraocular pressure.^2^

In conclusion, a patient with macular striae and acute onset myopia due to topiramate intake is presented.

## AUTHOR CONTRIBUTIONS

**Kishore Kumar Vatwani:** Conceptualization; data curation; investigation; methodology; project administration; resources; supervision; visualization; writing – original draft; writing – review and editing. **Koushik Tripathy:** Conceptualization; data curation; formal analysis; investigation; methodology; project administration; resources; software; supervision; writing – original draft; writing – review and editing. **Rohit Agarwal:** Data curation; formal analysis; investigation; methodology; project administration; resources; supervision; visualization; writing – original draft; writing – review and editing. **Gopal Bandyopadhyay:** Formal analysis; investigation; methodology; project administration; resources; supervision; visualization; writing – review and editing.

## FUNDING INFORMATION

No financial support was received for this submission.

## CONFLICT OF INTEREST STATEMENT

The authors have no conflict of interest with the submission.

## CONSENT

Written informed consent was obtained from the patient to publish this report in accordance with the journal's patient consent policy.

## Data Availability

The authors confirm that the data supporting the findings of this study are available within the article.
